# Supplementary material for: Anterior lateral motor cortex enables contextual decision-making via dynamic reconfiguration of local circuits
Source: Cell Rep. Author manuscript; Available in PMC 2026 Jul 30. (PMC13419430; doi:10.1016/j.celrep.2026.117456)
Supplement: 1 [file NIHMS2194659-supplement-1.pdf]

Cell Reports, Volume *45*

Supplemental information

Anterior lateral motor cortex enables contextual decision-making via dynamic reconfiguration of local circuits

Jia Shen, Nuttida Rungratsameetaweemana, Prayshita Sharma, Darcy S. Peterka, Herbert Zheng Wu, and Michael N. Shadlen

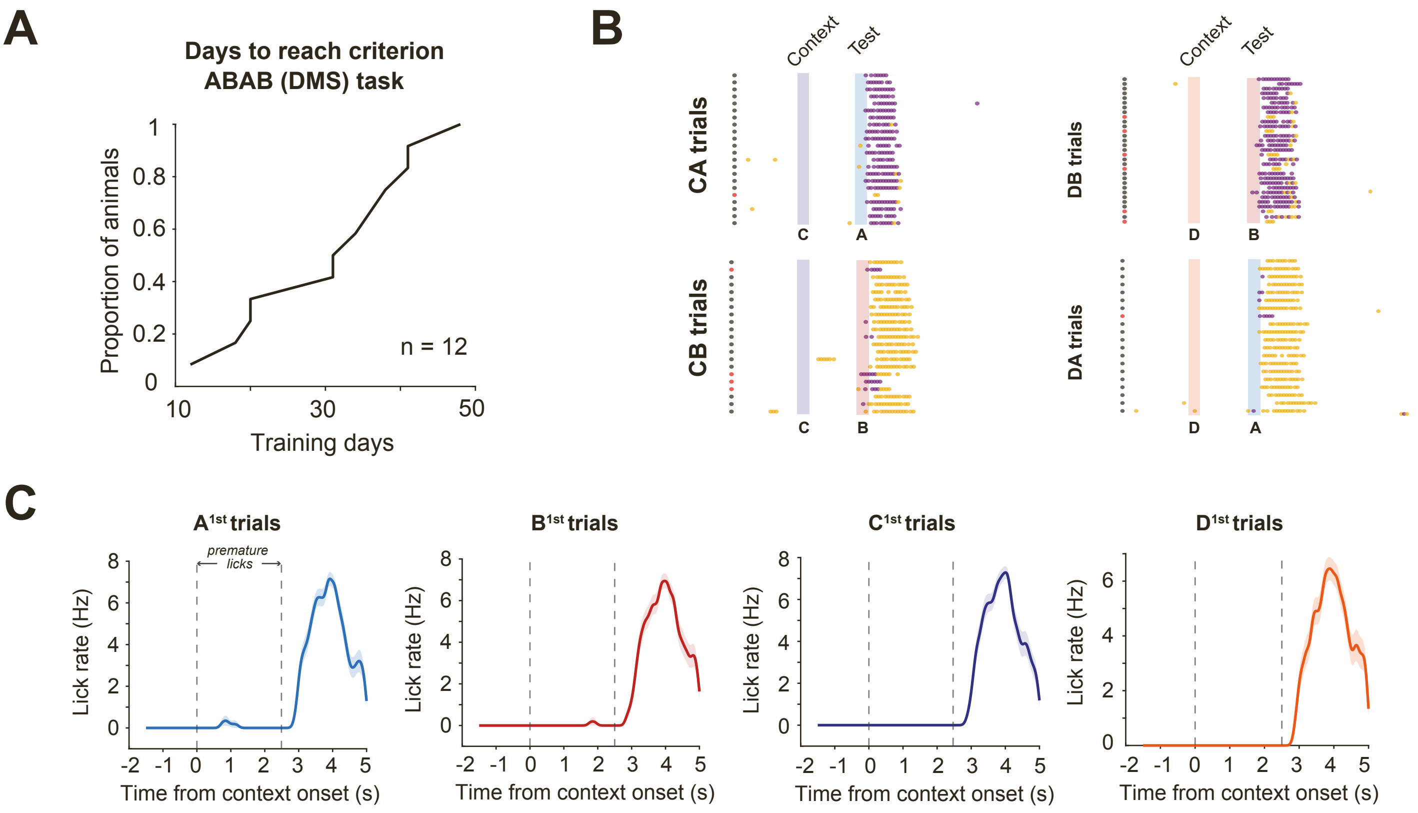

Figure S1. **Training time and licking behavior during the CDAB task**

(A) Days required to reach criterion performance during training on the ABAB task (n = 12, 30.8 ± 3.2 days).

(B) Example behavioral session showing licking timestamps. Each row represents one trial. Black marks indicate correct trials and red marks indicate error trials. Purple and yellow ticks denote licks to the left and right ports, respectively.

(C) Example lick-rate traces across the four context odor conditions. Dashed lines indicate the onset of the context odor and test odor. Premature licks were defined as licks occurring before test odor onset. Lick rates did not differ across context odor conditions (Kruskal–Wallis test, n.s., $p > 0.05$).

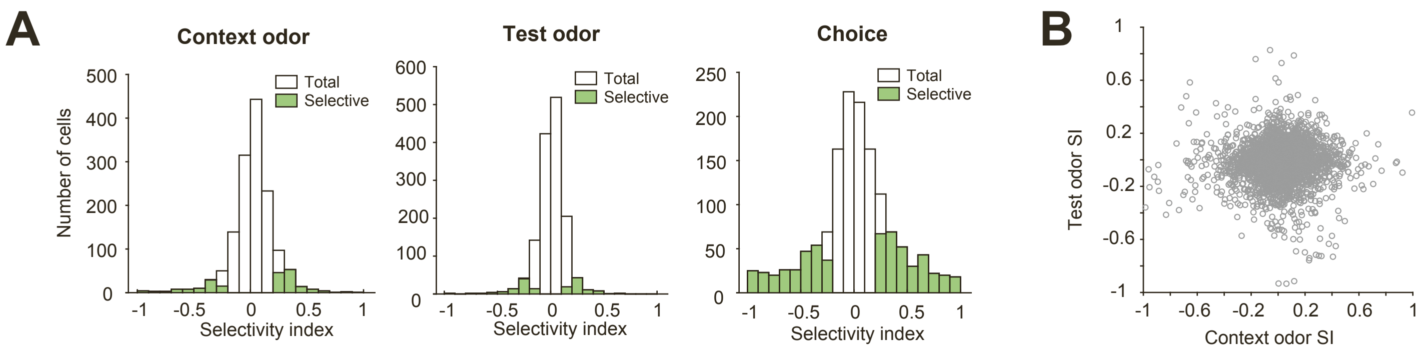

Figure S2. Distribution of context, test odor, and choice selectivity among ALM neurons

(A) Summary of the selectivity indices for each functional cell type in the CDAB task.

(B) Relationship between selectivity indices for context odor and test odor across ALM neurons (Pearson's correlation r = 0.03, p = 0.10).

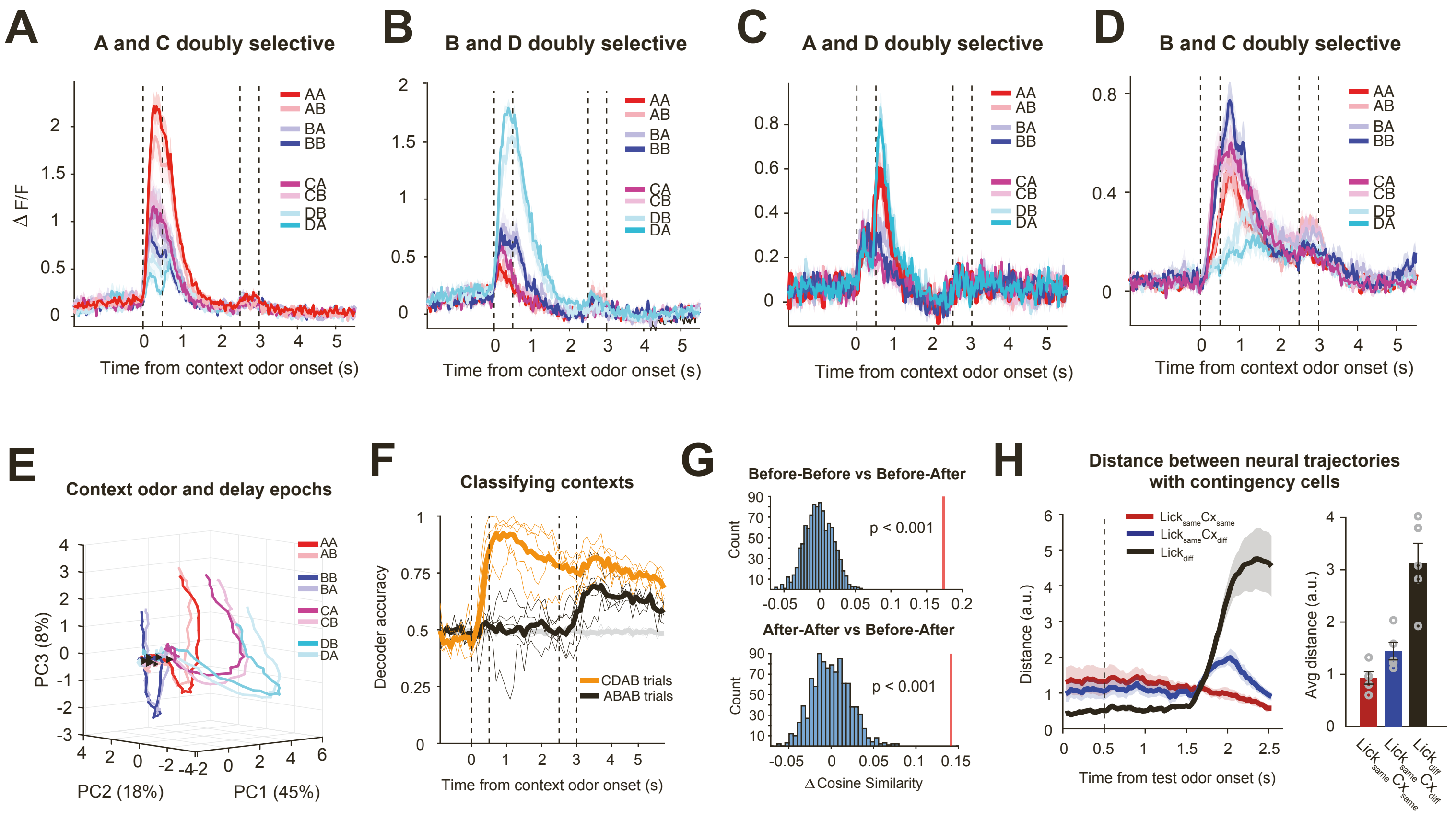

Figure S3. Decoding and population analyses reveal distinct neural representations of context odors

(A–D) Trial type-averaged Ca^{2+} responses from example doubly selective neurons corresponding to the four neurons shown in Figure 4D–G. Panels (A) and (B) show neurons selective for pairs of first odors that signal the same context (A^{1st} with C^{1st}; B^{1st} with D^{1st}). Panels (C) and (D) show neurons selective for pairs of first odors that signal opposing contexts (A^{1st} with D^{1st}; B^{1st} with C^{1st}).

(E) Neural trajectories in a 3D state space defined by the first three principal components, illustrating the divergence of activity associated with different context odors. Triangles indicate test odor offset.

(F) Cross-task decoding of context. SVM classifiers trained on CDAB trials were used to decode context in ABAB trials (black). Shuffled control performance is shown in gray and cross-validated decoder accuracy in CDAB trials is shown in orange.

(G) Monte Carlo permutation test assessing the cosine similarity between decoder weight vectors before and after test odor onset. Similarity between time points spanning the test odor onset (before-after) is significantly lower than similarity between time points within the same temporal block (before-before or after-after).

(H) Euclidean distance between PCA neural trajectories for three trial groups: trials with the same lick direction and context (red), trials with the same lick direction but different context (blue), and trials with different lick directions (black).

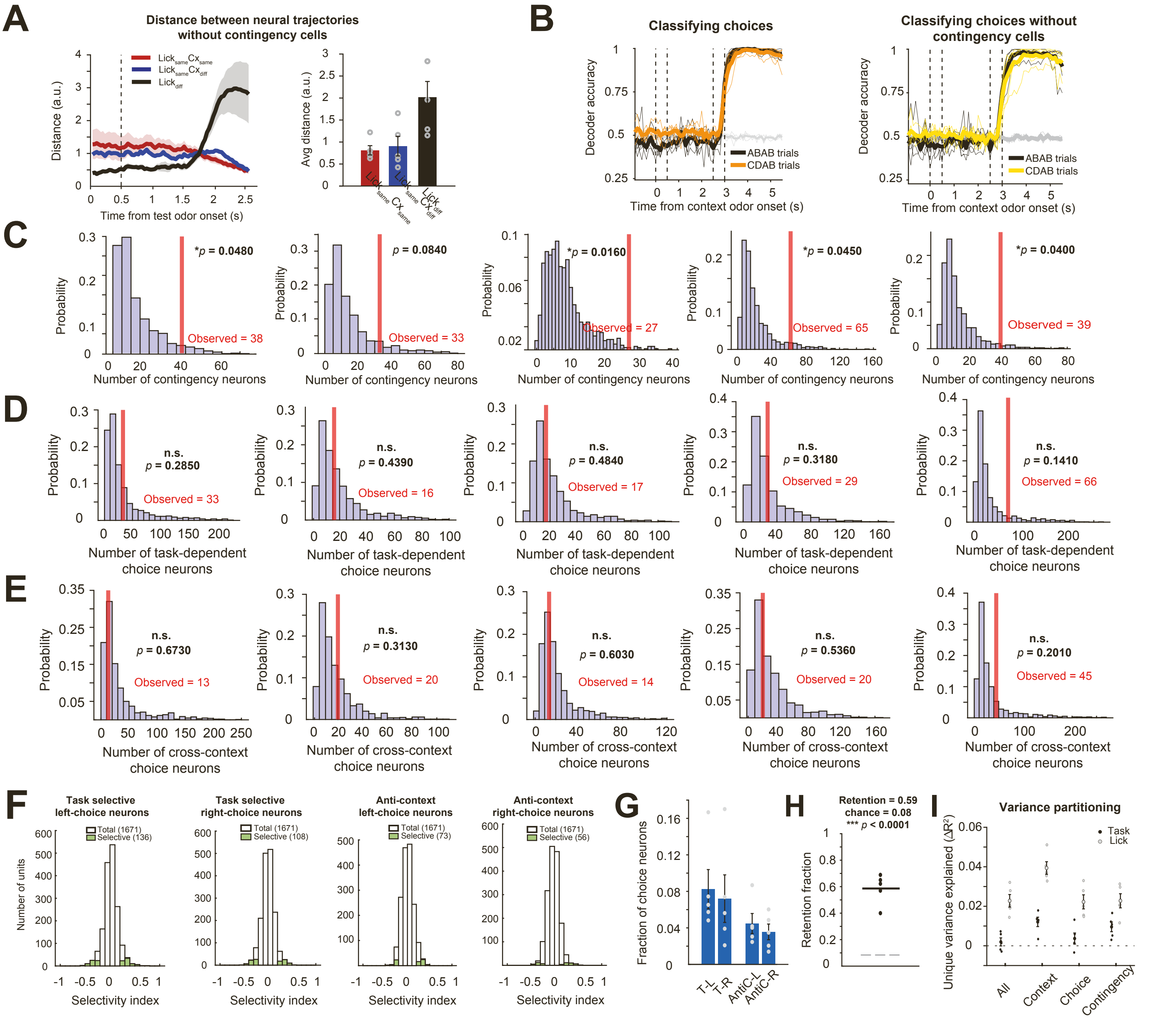

Figure S4. Analyses related to contingency neurons

(A) Euclidean distance between the PCA neural trajectories after removing contingency neurons. Distances are shown for three groups: trials with the same lick direction and context (red), trials with the same lick direction but different context (blue), and trials with different lick directions (black).

(B) Choice decoding using SVM classifiers trained on ABAB trials. Decoder performance is shown when contingency neurons are included (left) and after removing contingency neurons (right).

(C-E) Permutation test assessing the number of contingency neurons (C), task-dependent choice neuron (D), and cross-context choice neurons (E). Histograms show the distribution obtained from 1,000 simulated iterations for each animal; the red line indicates the observed number of contingency neurons in the data (*p < 0.05).

(F) Distribution of Selectivity Index values for task-dependent and anti-context choice neurons.

(G) Percentage of each type of choice neurons.

(H) Retention of contingency-selective neurons after removing licking-related activity. For each animal, contingency neurons were re-identified after regressing out licking-related variance from neural activity. Points indicate the fraction of contingency neurons retained after regression for individual animals (n = 5). The solid line indicates the mean retention across animals, and the dashed line indicates the overlap expected by chance.

(I) Variance partitioning of neural activity using cross-validated encoding models (Musall et al.[35]). Unique variance explained by task variables (black) and licking variables (gray) was quantified using ridge-regularized encoding models with time-resolved regressors. For each animal, median ΔR^2 values were computed across neurons within each group (all neurons, context-selective neurons, choice-selective neurons, and contingency neurons). Points indicate individual animals; larger symbols and error bars indicate mean ± s.e.m. across animals.
